# Does the Immune System Naturally Protect Against Cancer?

**DOI:** 10.3389/fimmu.2014.00197

**Published:** 2014-05-12

**Authors:** Alexandre Corthay

**Affiliations:** ^1^Tumor Immunology Group, Department of Pathology, Oslo University Hospital Rikshospitalet, Oslo, Norway; ^2^Department of Biosciences, University of Oslo, Oslo, Norway; ^3^Centre for Immune Regulation, University of Oslo, Oslo, Norway

**Keywords:** cancer immunosurveillance, primary immunodeficiency, cancer risk, organ transplantation, immunosuppressive drugs, HIV, NKG2D, checkpoint blockade

## Abstract

The importance of the immune system in conferring protection against pathogens like viruses, bacteria, and parasitic worms is well established. In contrast, there is a long-lasting debate on whether cancer prevention is a primary function of the immune system. The concept of immunological surveillance of cancer was developed by Lewis Thomas and Frank Macfarlane Burnet more than 50 years ago. We are still lacking convincing data illustrating immunological eradication of precancerous lesions *in vivo*. Here, I present eight types of evidence in support of the cancer immunosurveillance hypothesis. First, primary immunodeficiency in mice and humans is associated with increased cancer risk. Second, organ transplant recipients, who are treated with immunosuppressive drugs, are more prone to cancer development. Third, acquired immunodeficiency due to infection by human immunodeficiency virus (HIV-1) leads to elevated risk of cancer. Fourth, the quantity and quality of the immune cell infiltrate found in human primary tumors represent an independent prognostic factor for patient survival. Fifth, cancer cells harbor mutations in protein-coding genes that are specifically recognized by the adaptive immune system. Sixth, cancer cells selectively accumulate mutations to evade immune destruction (“immunoediting”). Seventh, lymphocytes bearing the NKG2D receptor are able to recognize and eliminate stressed premalignant cells. Eighth, a promising strategy to treat cancer consists in potentiating the naturally occurring immune response of the patient, through blockade of the immune checkpoint molecules CTLA-4, PD-1, or PD-L1. Thus, there are compelling pieces of evidence that a primary function of the immune system is to confer protection against cancer.

## Introduction

Lewis Thomas and Frank Macfarlane Burnet proposed the concept of immunological surveillance of cancer more than five decades ago (1–4). It was defined by Burnet as follows: “In large long-lived animals, like most of the warm-blooded vertebrates, inheritable genetic changes must be common in somatic cells and a proportion of these changes will represent a step toward malignancy. It is an evolutionary necessity that there should be some mechanism for eliminating or inactivating such potentially dangerous mutant cells and it is postulated that this mechanism is of immunological character” (1). More than 50 years after Burnet proposed his theory, the immunological scientific community remains largely divided with both proponents [e.g., Ref. (5, 6)] and opponents [e.g., Ref. (7, 8)] of the cancer immunosurveillance hypothesis. In fact, an opposite and very influential concept was proposed in 2001 by Frances Balkwill and Alberto Mantovani, who suggested that inflammatory immune cells and cytokines found in tumors may promote rather than suppress tumor growth (9, 10). Although, we are currently lacking convincing data illustrating immunological eradication of precancerous lesions *in vivo*, there are strong indications that a primary function of the immune system is indeed to prevent cancer. Here, I present eight types of evidence in support of the cancer immunosurveillance hypothesis.

## Primary Immunodeficiency in Humans and Mice is Associated with Increased Cancer Risk

As Burnet himself pointed out, an implication of the cancer immunosurveillance hypothesis is that immunodeficiency should be associated with increased likelihood of neoplasia (1). Immunodeficiencies can be divided in two main types: primary (inborn) immunodeficiencies, which are caused by genetic defects and whose incidence is approximately 1:10,000 births; and secondary immunodeficiencies, which are induced by immunosuppressive medication or viral infection and which are much more common. In accordance with Burnet’s prediction, severe primary immunodeficiencies have been reported to be associated with increased risk of malignancy (11–14). For instance, patients with defective humoral immunity due to common variable immunodeficiency (CVID) had increased incidence of lymphoma and epithelial tumors of the stomach, breast, bladder, and cervix (12, 15). Selective immunoglobulin A (IgA) deficiency was associated with a high incidence of gastric carcinomas (15). Moreover, patients with X-linked immunodeficiency with hyper-IgM, caused by mutations in the CD40 ligand molecule, had a high incidence of tumors of the pancreas and liver (16). However, it remains unclear to what extent primary immunodeficiency in humans leads to increased cancer development, due to the relatively low number of patients investigated.

Gene-targeted mice, which selectively lack key components of the immune system have been extensively used to experimentally test the effect of well-defined primary immunodeficiencies on cancer development [reviewed in Ref. (17)]. Mice which lacked both T and B cells, due to a deficiency in the recombination-activating gene 2 (RAG2), were more susceptible to spontaneous and carcinogen-induced carcinomas (18). Mice lacking γδ T cells were highly susceptible to multiple regimens of cutaneous carcinogenesis (19). The cytokines interferon-α/β (IFN-α/β) and IFN-γ were shown to protect mice against spontaneous and carcinogen-induced malignancy (18, 20–22). Moreover, the molecule perforin, which is used by cytotoxic lymphocytes to kill target cells, was reported to be important for surveillance of spontaneous lymphoma (23). Collectively, the human and mouse data reveal a consistent association between primary immunodeficiency and increased incidence of various types of cancer.

## Organ Transplant Recipients are More Prone to Cancer Development

A breakthrough in organ transplantation was the discovery of immunosuppressive drugs such as cyclosporine A, which prevent organ rejection by the adaptive immune system (24). Immunosuppressive medication is now standard treatment after organ transplantation. Life-long treatment of thousands of transplanted patients with immunosuppressive drugs was defined by Thomas as a “human experiment” to test the cancer immunosurveillance hypothesis (4). Already in 1973, an international registry-based study of renal-transplant recipients from 30 countries revealed that transplantation was associated with increased risk of developing cancer, in particular lymphoma (25). A large cohort investigation of cancer risk after organ transplantation was performed in the Nordic countries, in homogeneous populations with well-documented cancer incidence, on nearly 6000 kidney recipients (26). A two to fivefold excess risk was reported for cancers of the colon, larynx, lung, bladder, prostate, and testis. Strikingly high risks, 10-fold to 30-fold above normally expected levels, were observed for cancers of the lip, skin (non-melanoma), kidney, endocrine glands, cervix, and for non-Hodgkin’s lymphoma (26). Another large study of kidney transplantation in 200,000 patients from 42 countries reported that the risk of developing lymphoma was 12-fold higher for transplant recipients than that in a matched non-transplanted population (27). Notably, the majority of posttransplant lymphomas were associated with infection with Epstein–Barr virus (EBV), which primarily infects B cells and is known to cause B cell transformation (28). Thus, most lymphomas arising in transplant patients were likely to be a secondary event resulting from reduced antiviral immunity, rather than a direct effect of reduced antitumor immunity. However, lymphomas not associated with EBV infection have also been reported after transplantation (29). An investigation of 175,000 solid organ transplants in the USA revealed that increased cancer risk occurred not only after kidney transplantation but also after liver, heart, and lung transplantation (30). Risk was increased for 32 different malignancies, some related to known infections (e.g., anal cancer and Kaposi sarcoma) and others unrelated to infections (e.g., lung cancer and melanoma). The most common malignancies with elevated risk were non-Hodgkin lymphoma and cancers of the lungs (30).

Very high rates of non-melanoma skin cancers have been reported for Swedish (20–40%) and Australian (70%) populations 20 years after transplantation (31–33). Cutaneous types of human papillomaviruses have been suggested to be the cause of non-melanoma skin cancers such as squamous cell carcinoma in immunosuppressed patients, but the epidemiological pieces of evidence remain inconsistent (34). Strikingly, non-melanoma skin tumors in the renal-transplant population of Queensland, Australia, were reported to arise predominantly on chronically sun-exposed skin (head, neck, and distal limbs), strongly suggesting a causative role of ultraviolet (UV) light rather than oncogenic viruses (33). Thus, life-long treatment of organ transplant recipients with immunosuppressive drugs leads to increased risk of developing many different types of cancer, some related to known infections and others unrelated.

## Immunosuppression Induced by Infection by Human Immunodeficiency Virus Type 1 leads to Elevated Risk for Cancer

The HIV-1 virus causes acquired immunodeficiency by selectively infecting and killing CD4^+^ T cells. Accordingly, HIV-infected patients, receiving or not antiviral treatments, possess reduced levels of CD4^+^ T cells compared to non-infected individuals. HIV-infected individuals have elevated risk for cancer linked to oncogenic viruses such as Kaposi sarcoma (caused by human herpes virus 8), Hodgkin’s and non-Hodgkin’s lymphoma (EBV), anal and cervical cancer (human papilloma virus), and liver cancer (hepatitis B and C viruses). Kaposi sarcoma, non-Hodgkin’s lymphoma and cervical cancer are particularly frequent and are considered as acquired immunodeficiency syndrome (AIDS)-defining cancers (35). However, several cancers that are not linked to oncogenic viruses, like lung cancer and multiple myeloma, are also more frequent in patients with HIV (35, 36). Lung cancer is the most common non-AIDS-defining cancer and a leading cause of mortality among HIV-infected individuals (37). For the majority of patients with lung cancer, malignant transformation is known to be caused by carcinogens present in cigarette smoke. Higher smoking rates have been reported for HIV-infected populations. After controlling for potential confounders including smoking, a large cohort study of veterans (with 37,000 HIV-infected patients and 75,000 healthy controls) concluded that HIV was an independent risk factor for incident lung cancer (37). Importantly, cancer incidence in HIV-infected individuals was found to be inversely related to CD4^+^ T cell counts in blood, which supports the association between immunosuppression and increased cancer risk (38). For instance, the risk of lung cancer was doubled by CD4^+^ T counts in the range of 350–499 cells per microliter blood compared to normal counts ≥500, and continued to increase as the CD4^+^ T cell count fell (38). Thus, acquired immunodeficiency by HIV infection, which selectively depletes CD4^+^ T cells, leads to increased risk of developing many different types of cancer, some related to known infections, and others unrelated.

## Quantity and Quality of the Immune Cell Infiltrate in Human Primary Tumors Represent an Independent Prognostic Factor for Patient Survival

All solid tumors are infiltrated by a variety of immune cells. For many types of human cancers, an association has been reported between the type, density, and location of immune cells within the primary tumor and the clinical outcome [reviewed in Ref. (39)]. The number of intratumoral CD3^+^ T cells was shown to positively correlate with longer survival of patients with epithelial ovarian and colorectal cancers (40, 41). A high number of stromal CD4^+^ T cells were found to represent an independent positive prognostic factor in non-small cell lung cancer (42). Tumor-infiltrating CD8^+^ cytotoxic T cells were shown to predict clinical outcome in colon, lung, and breast cancers (42–45). Concurrent infiltration by both CD4^+^ and CD8^+^ T cells was reported to represent a favorable prognostic factor in esophageal squamous cell carcinoma and non-small cell lung cancer, suggesting that both cell types cooperate to fight cancer (46, 47). Among all CD4^+^ T cell subsets, Th1 cells seem to be particularly advantageous, as reported for colorectal, liver, and breast cancers (39, 40, 48, 49). In patients with gastrointestinal stromal tumors (GIST), the intratumoral density of CD3^+^ T cells and NKp46^+^ natural killer (NK) cells were found to represent two independent prognostic factors for progression-free survival (50). Notably, NK and T cells were detected in distinct areas of tumor sections, suggesting that both cell types contributed independently to GIST immunosurveillance (50). Furthermore, a high tumor infiltration by CD68^+^ macrophages was associated with prolonged survival in prostate, lung, and colon cancers (43, 51–54). Thus, for various types of human cancers, the quantity and the quality of the immune response within the primary tumor appear to represent an independent predictor for patient survival. This correlation between immunological data and clinical outcome strongly suggests that the immune system of the patient had naturally mounted an antitumor immune response before any treatment had started. The efficiency of this response presumably varies from patient to patient, thereby critically influencing survival.

## Cancer Cells Harbor Mutations in Protein-Coding Genes that are Specifically Recognized by the Adaptive Immune System

Cancer cells originate from normal cells that have accumulated “driver” mutations, which either activate oncogenes by dominant gain of function or inactivate tumor suppressor genes by recessive loss of function. A typical tumor contains two to eight of these driver mutations (55). Cancer cells also accumulate “passenger” mutations, which do not contribute to tumorigenesis. Genome-wide sequencing studies have provided detailed information about somatic mutations in various types of cancers. For common solid tumors such as breast, colon, brain, and pancreas cancers, an average of 30–60 non-synonymous mutations in protein-coding genes was observed (56–59). Most of these mutations (95%) were single-nucleotide substitutions, whereas the remainder was deletions or insertions (55). Metastatic melanoma and non-small cell lung carcinoma, which represent two types of cancers caused by potent mutagens (UV light and cigarette smoke, respectively), had a higher mutation rate with ~150 mutations per tumor (60, 61). Pediatric tumors and leukemias had the fewest mutations with ~10 mutations per tumor on average (55). Thus, it is now established that tumor cells in most cancer types harbor numerous non-synonymous mutations in protein-coding genes.

Driver and passenger mutations, which alter the normal amino acid sequence of proteins, may potentially be recognized by the adaptive immune system. A number of studies have revealed that tumor-specific antigens created by mutations can be recognized either by the T cells or the B cells of the patient. For instance in melanoma, CD4^+^ T cells were found that recognized a tumor-specific antigen generated by a non-synonymous point mutation in the gene coding for triosephosphate isomerase (62). Another antigen recognized by CD4^+^ T cells in melanoma had been generated by a chromosomal rearrangement resulting in a fusion of a low density lipid receptor gene with a fucosyltransferase gene (63). In colorectal cancer with microsatellite instability phenotype, CD4^+^ T cells were identified that recognized a frameshift mutation in the transforming growth factor β receptor II (TGFβRII) (64). In a melanoma patient, the tumor suppressor p16^INK4a^ with a point mutation was specifically recognized by cytotoxic CD8^+^ T cells (65). In non-small cell lung cancer, several CD8^+^ T cell epitopes created by point mutations have been reported (66–68). Moreover, in chronic myeloid leukemia, cytotoxic CD8^+^ T cells specific for a BCR-ABL fusion protein (resulting from the fusion of BCR and ABL genes) were found (69). Tumor-specific IgG antibodies are common in the serum of cancer patients, as revealed by serological identification of antigens by recombinant expression cloning (SEREX) technology (70). This powerful method has allowed the identification of over 2000 tumor antigens recognized by autologous IgG, including the p53 tumor suppressor modified by a point mutation (71). Collectively, these studies demonstrate that the adaptive immune system is able to detect cancer by specifically recognizing the mutated proteins of the malignant cells.

## Cancer Cells Selectively Accumulate Mutations to Evade Immune Destruction

Recognition of cancer cells by tumor-specific CD8^+^ T cells is achieved by the presentation of antigenic peptides from mutated proteins on major histocompatibility complex (MHC) class I molecules on the surface of cancer cells. In order to avoid recognition and the resulting elimination by CD8^+^ T cells, cancer cells often mutate key genes of the MHC class I antigen presentation pathway. Downregulation of surface MHC class I molecules is a common feature of human cancer cells [reviewed in Ref. (72)]. Several mechanisms have been reported, including mutations in the β2-microglobulin gene, which is required for MHC class I molecule expression on the cell surface (73, 74). MHC haplotype loss in various human tumors was shown to be caused by complete or partial loss of chromosome 6, which harbor all MHC class I and class II genes (except for β2-microglobulin) (75). On the basis of its mutation pattern in cancer cells, β2-microglobulin was recently included in a list of 74 tumor suppressor genes (55). A recent study analyzed somatic point mutations in exon sequences from 4742 human cancers across 21 cancer types (76). Based on mutation frequency and pattern, 254 “cancer genes” were identified, including four genes belonging to the MHC class I antigen presentation pathway (β2-microglobulin, HLA-A, HLA-B, and TAP1), as well as the CD1D gene, which is involved in the presentation of lipid antigens to NK T cells (76). Hence, several mutations frequently observed in cancer cells are likely to result from selective pressure to evade the immune attack, in particular by cytotoxic CD8^+^ T cells and NK T cells.

Another strategy used by cancer cells to avoid the immune response consists of secreting immunosuppressive cytokines such as transforming growth factor β (TGF-β) and interleukin 10 (IL-10). In contrast to normal cells, which produce very little, malignant cells often secrete large amounts of TGF-β and IL-10 [reviewed in Ref. (77)]. Both cytokines have various effects on non-transformed cells present in the tumor mass, most notably the inhibition of immune cell functions. For several types of cancers, elevated serum levels of TGF-β or IL-10 have been reported to be associated with worse prognosis [reviewed in Ref. (77)]. Surprisingly, TGF-β can function both as a tumor suppressor and a tumor promoter, this duality being known as the TGF-β paradox. In early stage tumors, TGF-β is a potent inducer of growth arrest. In advanced stage malignant cells, TGF-β signaling pathways are severely dysregulated, and TGF-β promotes tumor growth [reviewed in Ref. (78)]. Thus, cancer cells often produce abnormally high levels of immunosuppressive cytokines, which strongly suggests that dampening immunity is a prerequisite for tumor growth.

Experiments with immunodeficient mice have demonstrated that the immune system may exert a strong selective pressure on the cancer cells. By using the chemical carcinogen methylcholanthrene, sarcomas were induced either in wild-type mice or in RAG2-deficient mice, which lack both T and B cells (18). When transplanted into RAG2-deficient mice, all sarcomas grew progressively with equivalent kinetics. In contrast, when the tumor cells were injected into immunocompetent wild-type hosts, all sarcomas from wild-type mice grew progressively, while 8 of 20 (40%) sarcomas from RAG2-deficient mice were rejected (18). These data strongly suggest that in wild-type mice, there was selection of tumor cells that were more capable of surviving in an immunocompetent host. This provides an explanation for the apparent paradox of tumor formation in immunologically intact individuals. Based on these findings, Robert Schreiber and coworkers introduced the term “cancer immunoediting,” which was further developed into a general theory, to describe the sculpting actions of the immune response on developing tumors in immunocompetent individuals (18, 79).

## Lymphocytes Bearing the NKG2D Receptor are Able to Recognize and Eliminate Stressed Premalignant Cells

NK cells are innate lymphocytes that can kill malignant or infected cells. All NK cells and some T cells express the NKG2D molecule on the cell surface. NKG2D is an activating receptor, which serves as a major recognition receptor for detection and elimination of transformed cells (80). The ligands for NKG2D are self proteins that are poorly expressed by normal resting cells but upregulated on the surface of stressed cells. NKG2D ligands in humans include MICA, MICB, and six different ULBP proteins (81). In mice, NKG2D ligands include MULT1, five isoforms of RAE-1, and three isoforms of the H60 proteins (82). In humans, cells that express NKG2D ligands may be recognized and killed by either NK cells or γδ T cells in a process called lymphoid stress surveillance (83).

NKG2D ligands were shown to be upregulated in normal cells after treatment with DNA-damaging agents like ionizing radiations and UV light (84). It was concluded that the DNA damage response, which was known to arrest the cell cycle and enhance DNA repair, may also participate in alerting the immune system to the presence of potentially dangerous cells (84). Several studies suggested that expression of NKG2D ligands on transformed cells may be directly induced by oncogenes. For example, the BCR-ABL fusion oncogene was reported to control the expression of MICA in chronic myelogenous leukemia cells at the posttranscriptional level (85). Activation of the Ras oncogene was shown to upregulate the expression of RAE-1α/β in mouse cells, and ULBP1–3 and MICA/B in human cells (86). In a recent study, surface upregulation of NKG2D ligands by human epithelial cells in response to UV irradiation, osmotic shock, or oxidative stress, was shown to depend on the activation of the epidermal growth factor receptor (EGFR) (87). The EGFR pathway is frequently dysregulated in human cancer and it was proposed that activation of EGFR may regulate the immunological visibility of stressed premalignant cells (87). Surprisingly, several isoforms of RAE-1, like RAE-1ε, were found to be expressed not only by cancer cells, but also by some normal proliferating cells such as fibroblasts (88). The E2F transcription factor, which controls cell cycle entry, was shown to regulate RAE-1ε expression. These data suggest that NKG2D-bearing lymphocytes may control the proliferation of both normal and malignant cells (88).

MICA and MICB were found to be expressed by many, but not all, freshly isolated carcinomas of the lung, breast, kidney, ovary, prostate, colon, and liver (89, 90). Moreover, *in vitro* studies revealed that MICA and MICB contributed to the lysis of hepatocellular carcinoma cells by NK cells (90). The importance of NKG2D for cancer immunosurveillance *in vivo* gained support from experiments showing that cancer cells transfected with NKG2D ligands and injected into mice were rapidly rejected by NK cells and by CD8^+^ T cells (91, 92). Moreover, neutralization of NKG2D with blocking monoclonal antibodies rendered mice more susceptible to carcinogen-induced fibrocarcinoma (93). Gene-targeted mice deficient for NKG2D were shown to be more susceptible to the *in situ* development of prostate adenocarcinoma and B cell lymphoma (94). In humans, an association has been reported between polymorphisms of the NKG2D gene and susceptibility of developing liver and cervix cancers, supporting a protective role of NKG2D against these malignancies (95, 96). Thus, the expression of stress-induced endogenous molecules associated with cell transformation is used by the immune system to recognize and eliminate premalignant cells in mice and humans.

## Promising Novel Strategy to Treat Cancer Consists in Potentiating the Naturally Occurring Immune Response of the Patient Through Blockade of Immune Checkpoint Molecules

Activation of a naïve T cell requires at least two signals: T cell receptor-mediated recognition of a cognate antigen (signal 1) and engagement of the costimulatory receptor CD28 (signal 2). Once activated, T cells upregulate on the cell surface two co-inhibitory molecules, cytotoxic T lymphocyte-associated antigen 4 (CTLA-4) and programed death 1 (PD-1). The function of these co-inhibitory molecules is to tightly regulate the immune response by containing excessive T cell activation. For the purpose of cancer immunotherapy, monoclonal antibodies have been generated to potentiate the ongoing antitumor immune response of the patient, through “immune checkpoint blockade” of CTLA-4, PD-1, or PD-1 ligand (PD-L1). The outcome of the initial clinical trials with these new treatments is remarkable (97).

In a phase 3, randomized trial, the CTLA-4 blocking antibody ipilimumab was shown to prolong survival of patients with previously treated metastatic melanoma by ~4 months (98). This was a breakthrough in the treatment of metastatic melanoma because no other therapy had previously been shown to prolong survival in a phase 3 controlled trial. Another phase 3 trial with previously untreated metastatic melanoma patients showed that the overall survival was significantly longer in the group receiving ipilimumab combined with the chemotherapy drug dacarbazine than in the group receiving dacarbazine plus placebo (11 vs. 9 months) (99). Moreover, higher survival rates after 3 years were observed in the ipilimumab–dacarbazine group compared to controls (21 vs. 12%) (99).

Although no phase 3 trial has yet been published based on PD-1 or PD-L1 blockade, phase 1 studies showed promising results. PD-1 checkpoint blockade was tested in a phase 1 trial on patients with several types of advanced cancer. Cumulative response rates (complete or partial responses) were 18% among patients with non-small cell lung cancer (14 of 76 patients), 28% among patients with melanoma (26 of 94 patients), and 27% among patients with renal-cell cancer (9 of 33 patients). Responses were durable, 20 of 31 responses lasting 1 year or more in patients with 1 year or more of follow-up (100). In a phase 1 trial with anti-PD-L1 blocking antibodies, an objective response (complete or partial response) was observed in 9 of 52 patients with melanoma, 2 of 17 with renal-cell cancer, and 5 of 49 with non-small cell lung cancer. Responses lasted for 1 year or more in 8 of 16 patients with at least 1 year of follow-up (101). Finally, combined treatment of advanced melanoma was performed with both anti-CTLA-4 and anti-PD-1 blocking antibodies in a phase 1 trial. The objective response rate for all 53 treated patients in the concurrent-regimen group was as high as 40% (102). Thus, immune checkpoint blockade represents a promising new strategy to treat advanced cancer in humans. The success of this approach, which is based on potentiating the ongoing, naturally occurring antitumor immune response of the patient, provides another piece of evidence that fighting cancer is indeed a primary function of the immune system.

## Concluding Remarks

As summarized in this review, the scientific literature over the past 50 years has provided strong support to the cancer immunosurveillance hypothesis. Thus, it appears that our immune system does not only naturally protect us against infectious non-self (pathogens) but also against malignant self (cancer). Many cell types belonging to both the innate (NK cells and macrophages) and the adaptive (T and B cells) immune systems seem to be involved in cancer control. Our current understanding on how the immune system fights cancer remains very fragmentary. There are pieces of evidence for two main strategies used by the immune system to distinguish cancer cells from normal cells. On one hand, the adaptive immune system recognizes altered (mutated) self proteins in malignant cells. On the other hand, NK cells and γδ T cells recognize stress-induced self molecules (NKG2D ligands) on transformed cells. Yet, cancer cells originate from normal cells and a main challenge for successful antitumor immunity is to restrain the destruction of normal cells (autoimmunity). In fact, a recent study suggested that autoimmune disease may occur as a result of an inaccurate antitumor immune response (103). Scleroderma is an autoimmune connective tissue disease in which patients make antibodies to a limited number of autoantigens, including the RNA polymerase III subunit, encoded by the POLR3A gene. In several patients who had both scleroderma and cancer, genetic alterations of the POLR3A locus were found in the malignant cells, suggesting that POLR3A mutations triggered an adaptive antitumor immune response, which cross-reacted with normal tissue, causing autoimmune disease (103).

## Conflict of Interest Statement

The author declares that the research was conducted in the absence of any commercial or financial relationships that could be construed as a potential conflict of interest.
